# Early events following bovine leukaemia virus infection in calves with different alleles of the major histocompatibility complex  DRB3 gene

**DOI:** 10.1186/s13567-019-0732-1

**Published:** 2020-01-13

**Authors:** Agustina Forletti, Claudia María Lützelschwab, Rosana Cepeda, Eduardo N. Esteban, Silvina Elena Gutiérrez

**Affiliations:** 1 Laboratorio de Virología, Facultad de Ciencias Veterinarias, Centro de Investigación Veterinaria de Tandil (CIVETAN-CONICET-CIC), Universidad Nacional del Centro de la Provincia de Buenos Aires (U.N.C.P.B.A.), Pinto 399, 7000 Tandil, Buenos Aires Argentina; 2 Consejo Nacional de Investigaciones Científicas y Técnicas (CONICET), Buenos Aires, Argentina; 3 Area de Bioestadística, Facultad de Ciencias Veterinarias, Instituto Multidisciplinario de Ecosistemas y Desarrollo Sustentable, Universidad Nacional del Centro de la Provincia de Buenos Aires (U.N.C.P.B.A.), Pinto 399, 7000 Tandil, Buenos Aires Argentina

## Abstract

Cattle maintaining a low proviral load (LPL) status after bovine leukaemia virus (BLV) infection have been recognized as BLV controllers and non-transmitters to uninfected cattle in experimental and natural conditions. LPL has been associated with host genetics, mainly with the BoLA class II DRB3 gene. The aim of this work was to study the kinetics of BLV and the host response in Holstein calves carrying different BoLA-DRB3 alleles. Twenty BLV-free calves were inoculated with infected lymphocytes. Two calves were maintained uninfected as controls. Proviral load, total leukocyte and lymphocyte counts, anti-BLVgp51 titres and BLVp24 expression levels were determined in blood samples at various times post-inoculation. The viral load peaked at 30 days post-inoculation (dpi) in all animals. The viral load decreased steadily from seroconversion (38 dpi) to the end of the study (178 dpi) in calves carrying a resistance-associated allele (*0902), while it was maintained at elevated levels in calves with *1501 or neutral alleles after seroconversion. Leukocyte and lymphocyte counts and BLVp24 expression did not significantly differ between genetic groups. Animals with < 20 proviral copies/30 ng of DNA at 178 dpi or < 200 proviral copies at 88 dpi were classified as LPL, while calves with levels above these limits were considered to have high proviral load (HPL) profiles. All six calves with the *1501 allele progressed to HPL, while LPL was attained by 6/7 (86%) and 2/6 (33%) of the calves with the *0902 and neutral alleles, respectively. One calf with both *0902 and *1501 developed LPL. This is the first report of experimental induction of the LPL profile in cattle.

## Introduction

Enzootic bovine leucosis (EBL), one of the most frequent neoplastic diseases of cattle, is caused by the exogenous retrovirus bovine leukaemia virus (BLV). BLV is the type species of the genus *Deltaretrovirus* in the Retroviridae family. This genus also includes pathogenic viruses of human and non-human primates (human and simian T-cell leukaemia viruses) that share biological and molecular characteristics with BLV [1].

Enzootic bovine leucosis is recognized by the World Organization for Animal Health as a disease of importance for the international trade of cattle [2]. Most countries from the European Union, including France, the United Kingdom, Germany and Spain, have been declared officially free of EBL [3], and the remaining members have a very low level of infected herds. In other countries, infection by BLV continues to grow, mainly due to the absence of nationwide programmes for controlling this disease. Examples of the latter group are Argentina, Japan, the United States of America and Canada [4–7].

The productive and economic impact of BLV infection is observed mainly in dairy herds. The main direct productivity loss is caused by death due to lymphosarcoma. In countries with modern dairy production systems and no control programme for EBL, the best estimate of the cumulative lymphoma incidence in BLV-infected cows is 1–2%. In high-incidence herds, this estimate may reach 5% [8]. Moreover, EBL has a negative effect on milk yield and leads to increased premature culling [9–11].

Bovine leukaemia virus infection is characterized by the iceberg principle, which is typical of many viral diseases. While nearly 70% of the infected cattle remain asymptomatic, one-third of the infected cattle develop a benign condition termed persistent lymphocytosis (PL), which is characterized by a permanent increase in the number of circulating B lymphocytes. Only a small percentage of infected cattle, usually less than 5%, develop neoplastic disease, which is invariably fatal [12].

Studies from our laboratory have shown that BLV-infected, haematologically normal cattle (i.e., those animals that do not develop PL) comprise two well-defined groups of individuals, characterized by different levels of proviral load in peripheral blood and titres of antibodies against the BLV major proteins. One of the phenotypes is defined by a high proviral load (HPL, > 100,000 BLV proviral copies/µg of DNA) in peripheral blood and high antibody titres against the 51 kDa envelope glycoprotein of BLV (BLVgp51). These cattle are indistinguishable from PL animals in terms of proviral load and antibody titres. The remaining non-PL animals harbour an exiguous number of infected lymphocytes in peripheral blood, almost undetectable by most molecular methods, including nested PCR and real-time PCR, and are hence termed low proviral load (LPL) cattle. These cattle develop low titres of antiviral antibodies against BLVgp51, while antibodies against the main core protein of BLV (BLVp24) are undetectable in a majority of LPL cattle or are present at very low titres [13]. As LPL cattle maintain their phenotype for prolonged periods of time without developing any haematologic or pathologic condition, it has been proposed that these animals are naturally resistant or have an intrinsic capability for controlling BLV replication. Furthermore, the fact that these animals have been proven to not transmit the BLV to uninfected cattle in experimental and natural conditions has important implications for the control of the infection and the disease [14–16].

It is believed that lymphosarcoma and the subclinical stage of PL are the result of a complex interplay between the virus and host. The genetic influence of the host on the resistance and susceptibility to the development of PL was mapped to the major histocompatibility complex (MHC) class II BoLA-DRB3 gene [17, 18]. The phenotypes of HPL and LPL in BLV-infected cattle are also strongly associated with BoLA-DRB3 gene polymorphisms. The HPL phenotype has been associated with the BoLA-DRB3*1501 allele, while the LPL phenotype has been associated with the BoLA DRB3*0902 and *1701 alleles [19]. The association of proviral load with the BoLA-DRB3 polymorphism in BLV-infected cattle has been subsequently confirmed by others and extended to other bovine breeds [20–23].

Identification of the factors and events leading to the development of HPL and LPL is essential for the reduction of the frequency of infected animals at the herd level. The present work was conducted to experimentally reproduce the phenotypes of HPL and LPL in calves and to study the dynamics of viral and host response within the 1st weeks post-infection in Holstein cattle carrying susceptibility- and resistance-associated BoLA-DRB3 alleles.

## Materials and methods

### Experimental animals

Twenty-two BLV-free, 6- to 9-month-old Holstein calves carrying specific BoLA-DRB3 alleles were selected. The absence of anti-BLV specific antibodies was determined by testing their serum samples by ELISA 108 [24], while the absence of the BLV provirus in DNA from peripheral blood leukocytes (PBLs) was assessed by conventional PCR [13]. Six of the experimental calves carried the BoLA-DRB3*1501 allele, while the other seven calves carried the BoLA-DRB3*0902 allele. In both cases, the calves were heterozygous, and the accompanying allele had no effect with regard to BLV dissemination (neutral allele). The control group was integrated by six calves carrying neutral (N) alleles. Calves from the *0902/N group were derived from 4 sires, while calves from the *1501/N and N/N groups were derived from 2 and 3 sires, respectively. One calf found to carry both the BoLA-DRB3*0902 and *1501 alleles was included separately in the study. Two of the animals were not inoculated and maintained as negative controls.

All procedures involving animals were performed in accordance with ethical standards and approved by the Animal Welfare Committee of the Facultad de Ciencias Veterinarias, Universidad Nacional del Centro de la Provincia de Buenos Aires, Argentina.

### Genotyping of BoLA DRB3 alleles

The presence of the BoLA-DRB3*0902 and *1501 alleles was determined by allele-specific real-time PCR as previously described [25]. Confirmation of the presence of specific alleles and identification of the accompanying alleles was performed by amplification and sequencing from both ends of a 284-bp fragment of the BoLA-DRB3 gene as previously described [26]. Allele assignment from the sequence data was performed using the script Haplofinder [27].

### Experimental inoculation with BLV and acquisition of samples

Blood was obtained in a heparinized syringe from a naturally BLV-infected adult cow with PL. The donor cow had 24 012 lymphocytes/mm^3^ of peripheral blood and 275 567 BLV copies per µg of DNA from PBLs. Sixty microliters of blood from the donor cow were diluted in 1 mL of phosphate-buffered saline (pH 7.2) and inoculated via subcutaneous injection in each of the experimental calves.

Blood samples from the experimental animals were obtained by jugular venipuncture before inoculation and at 3, 7, 14, 30, 38, 45, 61, 88 and 178 days post-inoculation (dpi).

### Absolute leukocyte and lymphocyte counts

The absolute leukocyte count in heparinized blood samples was determined using an automatic haematological analyser (Cell-Dyn 1400, Abbott Laboratories, Illinois, USA). Differential leukocyte counts were determined by visual observation of May–Gründwald–Giemsa-stained blood smears under a microscope at 100× magnification.

### Determination of the anti-BLV antibody titre

Plasma was obtained by centrifugation of heparinized blood samples. The presence of anti-BLVgp51 antibodies was determined by competitive ELISA 108 in the sequential samples obtained at all time points, and time to seroconversion was determined. ELISA 108 is a validated sensitive and specific enzyme-based immunoassay that detects antibodies against the conformational G epitope of BLVgp51 by competition with a monoclonal antibody (for more details, see Ref. [24]). Two-fold dilutions of plasma samples, starting at 1:25, were tested by ELISA 108 to determine the anti-BLVgp51 titres of antibodies.

### Quantification of the proviral load

Peripheral blood leukocytes were obtained from heparinized blood samples after lysis of erythrocytes with ammonium chloride solution (150 mM NH_4_Cl, 8 mM Na_2_CO_3_ and 6 mM EDTA, pH 7) and centrifugation (1000 × *g* for 7 min at 4 °C). DNA was extracted from PBLs using the Illustra Blood Genomic Prep Mini Spin Kit (GE Healthcare) and quantified by measuring the absorbance at 260 nm (Nanodrop, Thermo Fisher Scientific Inc., USA). Absolute quantification of the proviral load was carried out by real-time PCR (qPCR). The detailed procedure for amplification of a 59-bp fragment of the *pol* gene of BLV by qPCR has been described elsewhere [28, 29]. Amplification was carried out in an ABI 7500 Real-Time PCR System (Applied Biosystems) and was monitored by incorporation of SYBR Green^®^ dye. A standard curve was prepared with DNA obtained from the foetal lamb kidney cell line, which was persistently infected with four proviral copies of BLV per cell. The limit of detection of the method was 10 proviral copies per reaction (30 ng of DNA). Calculation of the proviral copy number of the samples was carried out by interpolation of the obtained Cq value within the calibration curve.

### Expression of BLVp24

The expression of the 24 kDa main core protein of BLV, BLVp24, was determined in cellular extracts obtained from fresh PBLs and in cultured mononuclear cells from each calf. Fresh PBLs were obtained from 3 mL of heparinized blood that was mixed with 11 mL of a cold ammonium chloride solution (150 mM NH4Cl, 8 mM Na_2_CO_3_, 6 mM EDTA) and then centrifuged (7 min at 1000 × *g* at 4 °C) to obtain the PBLs, which were then washed once with phosphate-buffered saline and stored at −20 °C until being analysed for BLVp24 expression. This procedure was carried out immediately after the blood was obtained from the animal, with the PBLs isolated within 30 min after blood collection to avoid the activation of BLV expression.

Blood mononuclear cells for culture were obtained by centrifugation on a Ficoll-Paque™ Plus gradient (GE Healthcare, Cat 17-1440-02) as previously described [30]. Cell viability was determined by the trypan blue exclusion method [31]. Peripheral blood mononuclear cell (PBMC) cultures were established at 5 × 10^6^ cells/mL in RPMI 1640 medium supplemented with 10% foetal calf serum and 10 µg/mL of concanavalin A at 37 °C in 5% CO_2_. After 18 to 20 h of culture, the cells were harvested by centrifugation (900 × *g* for 5 min at 4 °C) and washed once in phosphate-buffered saline.

Cell extracts from both fresh and cultured cells were prepared as previously described [32]. The total protein concentration in the cell extracts was determined by the method described by Bradford [33] using the Bio-Rad dye reagent and bovine serum albumin as the standard. The quantification of BLVp24 in cell extracts was carried out by a capture ELISA as previously described [30]. The results are expressed in nanograms of BLVp24 per milligram of total protein. The limit of detection of the capture ELISA was 11 ng/mL.

### Statistical analysis

Proviral load and BLVp24 expression data were log transformed for statistical analysis. The effects of BoLA-DRB3 alleles and time post-inoculation on the proviral load, leukocyte count and lymphocyte count and on the in vitro expression of BLVp24 were analysed by repeated measures ANOVA. The effect of BoLA-DRB3 alleles on time to seroconversion was analysed by ANOVA. Anti-BLVgp51 antibody titres were compared between the genetic groups by the Kruskal–Wallis test and Dunn’s multiple comparisons tests, while comparisons of titres between HPL and LPL phenotypes were performed by the Mann–Whitney test. In vitro expression of BLVp24 was analysed by ANOVA with proviral load as a covariate and dpi as a factor. Fisher’s exact test of independence was used to analyse the association between BoLA DRB3 genetics and the infection phenotype (HPL or LPL). Repeated measures ANOVA was also used to analyse the effect of the BLV phenotype and dpi on the in vitro expression of BLVp24. Differences were considered statistically significant at *p* < 0.05. Statistical analyses were conducted with PROC MIXED procedures in SAS v9.3 (SAS Institute Inc., Cary, NC, USA).

## Results

All experimental animals were negative for anti-BLV-specific antibodies and BLV proviral sequences in the blood sample obtained before the experimental inoculation. The animals maintained as negative controls retained their negative status throughout the study. At 60 dpi, tuberculin was inoculated into experimental calves, and 6 of them were positive. These animals were consequently removed from the trial at 90 dpi and sent to slaughter, as prescribed in the Argentinian legislation. The tuberculin-positive animals were found in both the inoculated (1 animal each for the 1501/N and N/N groups and 2 animals for the 902/N group) and non-inoculated (1 individual) groups. The last tuberculin-positive calf had the BoLA-DRB 902/1501 genotype. We did not find a significant association between the result of the tuberculin test and the BoLA-DRB3 genotype of the animals. However, before studying the effect of the BoLA-DRB3 genotype on the variables under study, we evaluated the effect of positivity in the tuberculin test on each variable. The viral and immunological parameters under study were not significantly affected by the reactivity to the tuberculin test [34].

**Figure 1:**
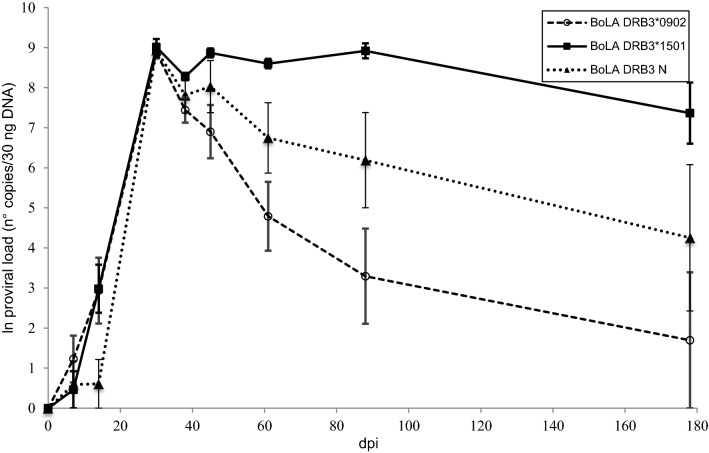
**Kinetics of the proviral load in calves experimentally infected with BLV.** Calves were experimentally inoculated with blood from an adult cow with PL. The proviral load was quantified by qPCR in samples of DNA extracted from PBLs from calves carrying different BoLA-DRB3 alleles. The data were log transformed before analysis. Mean values ± standard errors. *N* = 6 calves with BoLA DRB3*1501, *n* = 7 calves with BoLA DRB3*902 and *n* = 6 calves with neutral alleles.

Figure 1 shows the kinetics of the proviral load in the animals grouped according to the BoLA-DRB3 alleles. The BLV provirus was first detected at 7 dpi in only five of the experimental animals, although at very low levels (< 35 proviral copies/30 ng of DNA), while in the remaining animals, the provirus was first detected at 14 or 30 dpi. The model proposed to analyse the dependency of the proviral load in terms of BoLA genetics throughout the duration showed a significant interaction (*p* = 0.0029) between time and genetic group. Significant changes in proviral load were observed in all three groups between 14 and 30 dpi (*p* < 0.0001), reaching a peak at 30 dpi in all calves. There were no statistically significant differences in proviral load at 30 dpi between groups. A significant decrease in proviral load was detected at 38 dpi in calves with the *0902 allele (*p* < 0.0005) and in calves with N alleles (*p* < 0.01). At 45 dpi, the proviral load increased in calves carrying N or *1501 alleles, while in the group with the *0902 allele, the proviral load continued to decrease. In the latter group, the proviral load showed a steady decline until the end of the experiment. In calves with BoLA-DRB3 N alleles, the proviral load also decreased from 45 dpi until the end of the experiment. On the other hand, in the group carrying the *1501 allele, a third peak in the proviral load was reached at 88 dpi. Significant differences between groups were observed at 14 dpi (N/N was different from *0902/N and *1501/N, *p* < 0.05) and at 45 (*p* < 0.05), 61 (*p* < 0.005), 88 (*p* < 0.001) and 178 (*p* < 0.05) dpi between *1501/N and *0902/N.

**Figure 2:**
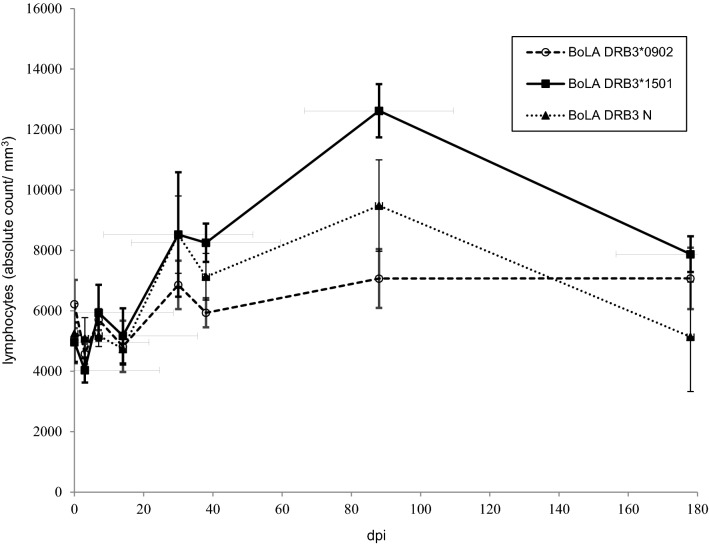
**Kinetics of the lymphocyte count in calves experimentally infected with BLV.** Calves were experimentally inoculated with blood from an adult cow with PL. Absolute leukocyte counts were determined in heparinized blood samples using an automatic haematological analyser. Differential leukocyte counts were determined by visual observation of May–Gründwald–Giemsa-stained blood smears. Mean values ± standard errors are depicted. *N* = 6 calves with BoLA DRB3*1501, *n* = 6 calves with BoLA DRB3*0902 and *n* = 6 calves with neutral alleles.

The variation in lymphocyte numbers over time did not significantly differ between groups (*p* = 0.26, Figure 2). While the leukocyte count was not significantly influenced by the BoLA-DRB3 alleles (*p* = 0.6717), this variable was influenced by dpi (*p* = 0.0011). A significant increase was detected at 30 dpi, while a significant decrease in lymphocyte numbers was observed between 88 and 178 dpi (*p* < 0.05).

**Figure 3:**
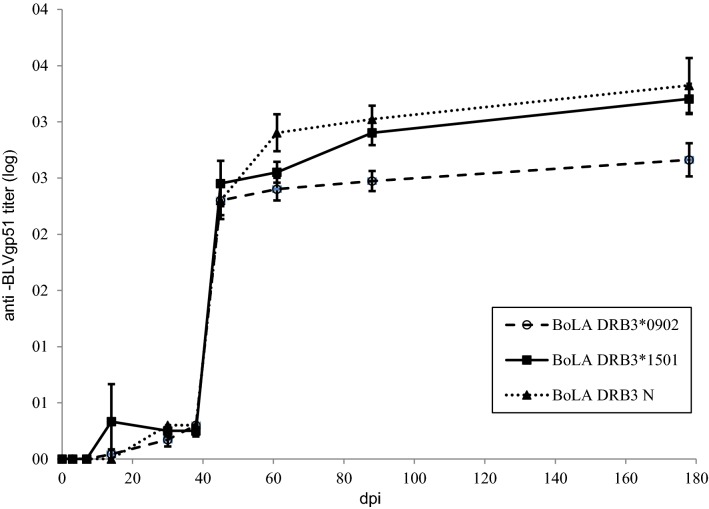
**Kinetics of the anti-BLVgp51 antibody titre in experimental calves.** Calves were experimentally inoculated with blood from an adult cow with PL. The antibody titre was determined by testing plasma samples by ELISA 108 in 18 calves carrying different BoLA DRB3 alleles. *N* = 6 calves with BoLA DRB3*1501, *n* = 7 calves with BoLA DRB3*902 and *n* = 5 calves with neutral alleles. The antibody titre was log transformed (mean ± standard error).

Anti-BLVgp51 antibodies were first detected between 14 and 45 dpi in all except one calf, which seroconverted at 88 dpi (this case was considered an outlier and excluded from the statistical analysis). In 12 animals, the provirus was first detected by PCR, while in the remaining seven calves, the antibodies and provirus were detected at the same dpi. Time to seroconversion did not significantly differ between genetic groups (*p* = 0.9479). The evolution of antibody titres in the three genetic groups is depicted in Figure 3. Anti-BLVgp51 antibody levels significantly increased at 45 dpi in all animals and were maintained at the same level in cattle carrying the *0902 allele. In calves carrying the *1501 allele or N alleles, antibody titres continued to increase over time and were on average higher in cattle carrying N alleles. At 88 and 178 dpi, the antibody titres in calves carrying the *0902 allele were on average 2.8 to 5.7 times lower than those in the other two groups; these differences were statistically significant (*p* < 0.05) when the *0902 animals were compared to animals carrying N alleles.

**Figure 4:**
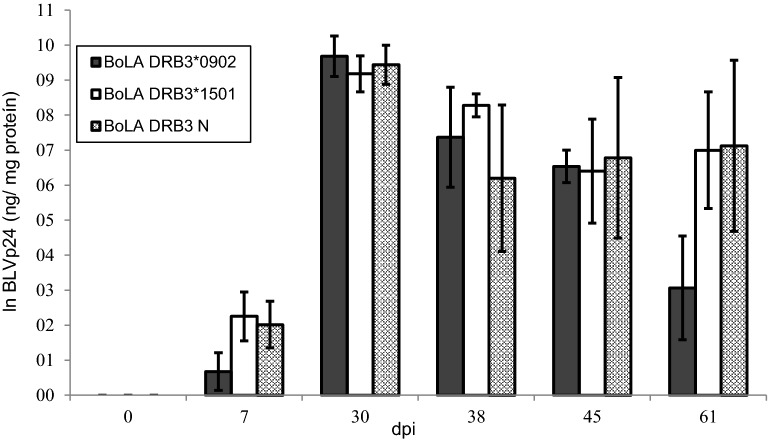
**BLVp24 expression in PBMC cultures of experimental calves.** Calves were experimentally inoculated with blood from an adult cow with PL at day 0. PBMCs were cultured in RPMI with foetal calf serum and concanavalin A for 18–20 h, and BLVp24 was quantified in cell extracts by a capture ELISA. *N* = 6 calves with the BoLA-DRB3*1501 allele, *n* = 7 calves with the BoLA-DRB3*0902 allele and *n* = 4 calves with neutral alleles. Values were log transformed (mean ± standard error)

BLVp24 levels were determined in fresh PBLs as well as in cultured mononuclear cells from the experimental animals and were used as an indicator of viral expression. Expression of this viral protein in fresh PBMCs was detected at very low levels (< 320 ng BLVp24/mg of total protein) in only four of the animals (2 from the group with the *0902 allele and two from the group with the *1501 allele) at 30 or 38 dpi. In contrast, high levels of BLVp24 were observed in the in vitro-cultivated cells from the animals. The expression of this protein in the three genetic groups is presented in Figure 4. BLV expression did not significantly vary between calves with different BoLA-DRB3 alleles (*p* = 0.2951), but it was influenced by dpi (*p* < 0.0001). A statistically significant increase in BLVp24 expression was observed between 7 and 30 dpi (*p* < 0.0001), while a decrease occurred at 38 dpi (*p* < 0.05).

When in vitro expression of BLVp24 was modelled using proviral load as a covariate and time post-inoculation as a factor (independent variable), it was found that both the proviral load (*p* = 0.0002) and time post-inoculation (*p* = 0.0004) were statistically significant.

The experimental calves were then classified according to the proviral load attained at the end of the experiment. Animals with < 20 proviral copies/30 ng of DNA at 178 dpi or < 200 proviral copies at 88 dpi were classified as having LPL, while calves with proviral load levels above these limits were considered to have HPL (Table 1). Six out of seven calves carrying the resistance-associated allele developed LPL, while all six animals carrying the susceptibility-associated allele developed HPL. The only calf with both the *0902 and *1501 alleles developed LPL. No significant association was found between reactivity in the tuberculin test and the infection profile of the animals [34]. In contrast, the association between the BLV infection profile and BoLA-DRB3 genetics was statistically significant (*p* < 0.01).

**Table 1:** **Distribution of calves experimentally inoculated with BLV according to their BoLA-DRB3 alleles and BLV infection profile established at 88 or 178** **dpi**

BLV infection profile	BoLA DRB3 alleles		
	*0902/N	*1501/N	N/N
HPL	1	6	4
LPL	6	0	2

HPL: high proviral load, LPL: low proviral load, N: neutral allele.

**Figure 5:**
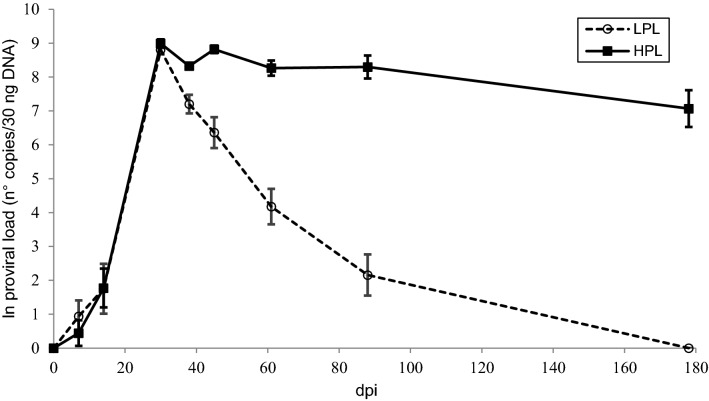
**Kinetics of the proviral load in calves that developed HPL and LPL.** Calves were experimentally inoculated with blood from an adult cow with PL at day 0. The proviral load was determined in peripheral blood by qPCR at various days post-inoculation (dpi). The data were log transformed before analysis. Mean values ± standard errors are shown. *N* = 11 calves with HPL and *n* = 9 calves with LPL.

**Figure 6:**
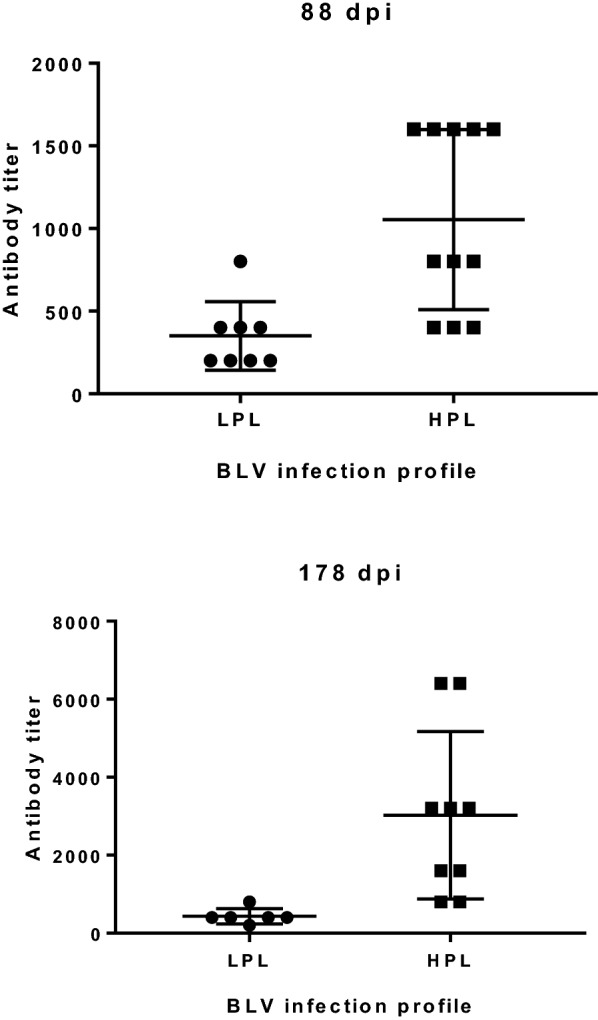
**Anti-BLVgp51 antibody titres in experimentally infected calves with LPL and HPL.** Anti-BLVgp51 antibody titres were determined in plasma samples by ELISA 108 at 88 and 178 dpi in experimental calves that developed LPL and HPL. *N* = 8 calves with LPL and *n* = 11 calves with HPL. Horizontal lines represent mean values, and error bars represent SDs.

**Figure 7:**
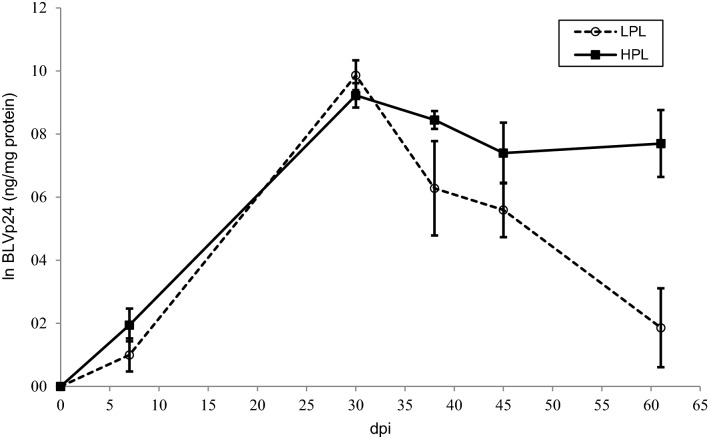
**BLVp24 expression in PBMC cultures of experimentally inoculated calves with HPL and LPL at different times post-inoculation.** PBMCs obtained at different times post-inoculation were cultured in RPMI with 10% foetal calf serum and concanavalin A (10 µg/mL) for 18–20 h. BLVp24 was quantified in cell extracts by a capture ELISA. Mean ± standard error values of log transformed quantities are shown. *N* = 10 calves with HPL and *n* = 8 calves with LPL.

The kinetics of the provirus significantly differed between HPL and LPL calves from 45 dpi until the end of the study (Figure 5). While proviral load was maintained at high levels in HPL animals, it decreased in LPL animals, reaching undetectable levels at 88 or 178 dpi. Statistically significant differences in the proviral load between HPL and LPL cattle were detected at all time points from 45 dpi (*p* < 0.005). The 95% confidence interval (α = 0.05) for the mean value of proviral load at 88 and 178 dpi was calculated for each group (Table 2). Antibody titres were significantly higher in HPL calves than in LPL calves at 88 and 178 dpi (*p* < 0.005) (Figure 6). BLVp24 expression was also compared between HPL and LPL animals at different time points after the experimental inoculation. The kinetics of BLV expression differed between the two groups (Figure 7, *p* < 0.05). BLVp24 was expressed at similar levels in HPL and LPL calves until 30 dpi. Then, a significant decrease (*p* < 0.05) in the level of expression was detected in LPL calves at 38 and 61 dpi, while in HPL calves, this parameter was maintained at high levels until 61 dpi. Statistically different values of BLVp24 expression between HPL and LPL animals were detected at 61 dpi (*p* < 0.05).

**Table 2:** **Ninety-five percent confidence interval (α = 0.05) for the mean proviral load at 88 and 178** **dpi for HPL and LPL animals**

Days post-inoculation	BLV infection profile	
	High proviral load	Low proviral load
88	6964.9 ± 2516.9	36.3 ± 55
178	3117.4 ± 1692.8	0 ± 0

Proviral load was quantified in peripheral blood by qPCR and is expressed as proviral copies/30 ng of DNA.

## Discussion

Bovine leukaemia virus-infected animals with the LPL phenotype have been proposed to be naturally resistant to BLV dissemination [15]. Knowledge regarding the causes and mechanisms that lead to the progression to HPL or LPL phenotypes is of paramount importance for elucidation of the virus–host relationship and to design and implement strategies to control the infection and disease in cattle populations. In previous studies, no differences were found in the biological and molecular properties of the viral strains from HPL and LPL cattle that could explain the different infection profiles [35–37]. On the other hand, the association between HPL and LPL phenotypes with polymorphisms at the BoLA-DRB3 gene indicates that host genetics is one of the main factors determining the outcome of BLV infection [19]. The current knowledge on HPL and LPL phenotypes has come from studies on naturally infected animals in which infection had been established for at least 6 months [13]; therefore, the early events leading to each of the phenotypes are unknown.

During the course of the experiment, six of the calves exhibited a positive reaction to the purified bovine tuberculin protein derivative when it was administered at 60 dpi for diagnostic purposes. These animals were sampled at 88 dpi for the last time and then sent to slaughter. The effect of *Mycobacterium bovis* infection on the virological and host immunological parameters evaluated in the present study was analysed using data from experimentally infected animals as well as data from naturally infected and BLV negative control calves (data not shown). None of the evaluated parameters were affected by infection with *M. bovis*. Moreover, although the number of analysed animals was limited, it was found that the reactivity to bovine tuberculin was not associated with any of the BoLA-DRB3 genotypes in the population studied [34].

To study the dynamics of BLV infection in cattle that developed HPL and LPL profiles, we selected cattle according to BoLA-DRB3-specific alleles that were previously shown to be associated with susceptibility and resistance to BLV dissemination [19]. In this experiment, all six animals carrying the susceptibility-associated allele BoLA DRB3*1501 developed HPL. Likewise, a high proportion (6/7) of the calves carrying the resistance-associated allele BoLA DRB3*0902 developed the LPL phenotype. A statistically significant association between the infection profile and BoLA-DRB3 genetics was determined, and consequently, the approach of inoculating BoLA-selected animals with BLV was successful for experimentally reproducing the expected phenotypes. The proportion of calves that developed the LPL phenotype in each of the genetic groups was quite similar to that observed in a previous study involving 230 naturally infected cattle from different herds. The fact that only one of the calves carrying the BoLA DRB3*0902 allele developed HPL can be explained by the high, but not complete, penetrance of the *0902 allele with regard to the development of the LPL profile [19]. These results confirm and extend previous findings on the association of BLV infection profiles with the BoLA-DRB3 genotype and constitute the first report on the experimental induction of the LPL phenotype.

The criteria for classifying infected animals into HPL or LPL profiles were originally established in naturally infected animals in which the proviral load was determined by semi-quantitative PCR. Cattle were classified as LPL when they had < 100 proviral copies/µg of DNA for at least 6 months [13]. In the present study, the criteria for defining the two profiles based on qPCR results were established according to the kinetics of the proviral load. This work should be considered a first approach towards the definition of BLV infection profiles based on quantitative levels of the proviral load determined by qPCR. Further studies with a greater number of naturally infected cattle should support these criteria.

In the present study, leukocyte and lymphocyte counts were not influenced by the BoLA-DRB3 genotype, although both parameters varied throughout the post-inoculation period. Transient lymphocytosis at the time of seroconversion was also observed after experimental BLV infection of sheep, and this response was independent of the type of virus inoculated (wild type or attenuated) [38–40].

It is believed that BLV is repressed in vivo at the transcriptional level and that the virus can be easily de-repressed upon in vitro cultivation for a few hours [41]. It seems that transcriptional repression of the virus is a mechanism that BLV has evolved to evade immune surveillance and to hence persist in its host and in nature. Based on this fact, it has been postulated that viral expression occurs in a subpopulation of infected cells, which are very efficiently killed by the immune system, and the viral transcripts or proteins are rarely detected by most methods [42, 43]. Our results, showing a low level of BLVp24 expression in vivo in only 4 of 20 experimental calves, may support the idea that cells expressing the BLV signal are rapidly removed by the immune system, most likely by cytotoxic T lymphocytes (CTLs). It is conceivable that a proportion of provirus-positive cells start to express Tax and are then rapidly killed by CTLs before expressing the structural proteins, as has been proposed for HTLV-1-infected cells [44]. This explanation may account for the low proportion of animals expressing a low level of the main core protein of BLV in vivo.

In the present study, differences in BLVp24 expression could not explain the distinctive kinetics of the proviral load between HPL and LPL cattle. Other more sensitive methods, such as qPCR for detecting transcripts or single-cell analysis, could, however, detect differences in viral expression between the groups.

The kinetic study of the proviral load shows that BLV replicates to the same extent independently of host genetics up to 30 dpi. The time to seroconversion did not differ between the genetic groups, which is consistent with the fact that the level of viral replication was similar between groups during the first 30 dpi. Then, and coincidently with seroconversion, differences in the level of proviral load were evident between the genetic groups. Based on our results, the LPL phenotype seems to be established from 38 dpi, immediately after seroconversion. The kinetics of the proviral load in HPL animals are similar to those reported in sheep experimentally inoculated with wild-type BLV [40]. Although calves seroconverted at the same time post-inoculation independently of the BoLA-DRB3 genetics, anti-BLVgp51 antibody titres in LPL calves were significantly lower than those in HPL calves, as described in naturally infected LPL cattle [13]. As the differences in proviral load in the different genetic groups began after 38 dpi, with sufficient time for the activation and expansion of the adaptive immune response effectors, it seems reasonable to assume that the control of viral propagation in LPL animals would be associated with a powerful cellular and/or humoral immune response. The underlying mechanisms may be related to the innate immune receptors that sense BLV and to the way in which these interactions contribute to the induction of a robust immune response that efficiently controls the viral load. In human immunodeficiency-1 (HIV-1) elite controllers, which maintain undetectable levels of HIV-1 and prevent the progression to acquired immunodeficiency syndrome, myeloid dendritic cells show unique antigen presenting properties associated with a distinct surface expression pattern of immunomodulatory leukocyte-immunoglobulin-like receptors (LILR) and a reduced ability to secrete pro-inflammatory cytokines [45]. These individuals exhibit a unique phenotype of CD8+ T cells, with high potential to expand upon exposure to the antigen and a striking capacity to eliminate HIV-1-infected cells [46]. Individuals infected with human T-cell leukaemia virus type 1 (HTLV-1) with a low HTLV-1 proviral load have been shown to express a core of at least nine genes encoding proteins involved in cell mediated lysis or antigen recognition, such as granzymes and granulolysin [47]. Investigation of differential host transcript expression between LPL and HPL cattle at the early phase of the infection would shed light on the immunological mechanisms involved in the control of the proviral load.

Usui et al. [48] showed increased expression of interferon-γ at the early phase of infection in experimentally infected sheep with impaired viral propagation, suggesting that cell-mediated immunity was involved in the suppression of viral propagation. However, it is not known whether the impaired propagation of the virus in these sheep is related to the animal MHC genetics. An elevated level of interferon-γ expression was also found in LPL cattle compared to HPL cattle [49]. A major function of BoLA class II molecules is to present processed peptide antigens to T cells to activate the adaptive immune response. Therefore, it is possible that the polymorphisms at the peptide-binding cleft in MHC class II molecules, responsible for the affinity of the bound peptides, influence the magnitude and extent of the elicited adaptive immune response and therefore the level of the proviral load. A genome-wide scan for single-nucleotide polymorphisms influencing the level of the proviral load in 800 BLV-infected cattle showed that all 24 significant associations were mapped to bovine chromosome 23, delimiting a region within the MHC [50]. Another conceivable mechanism underlying the control of the proviral load could be the action of other polymorphic genes within the MHC, acting independently or in linkage disequilibrium with BoLA-DRB3. Further research on the differential expression of transcripts in this experimental model of LPL progression should shed light on the events leading to the LPL profile.

## Abbreviations

LPL: low proviral load
BLV: bovine leukaemia virus
HPL: high proviral load
dpi: days post-inoculation
BoLA: bovine leukocyte antigen
EBL: enzootic bovine leukosis
PL: persistent lymphocytosis
BLVgp51: glycoprotein of 51 kDa from bovine leukaemia virus
BLVp24: main core protein of 24 kDa from bovine leukaemia virus
PCR: polymerase chain reaction
MHC: major histocompatibility complex
N: neutral allele
qPCR: real-time PCR
PBL: peripheral blood leukocyte
PBMC: peripheral blood mononuclear cell
ANOVA: analysis of variance
CTLs: cytotoxic T lymphocytes
